# Undergraduate medical education and Covid-19: engaged but abstract

**DOI:** 10.1080/10872981.2020.1781379

**Published:** 2020-06-16

**Authors:** David Hammond, Christina Louca, Laura Leeves, Sanketh Rampes

**Affiliations:** Faculty of Life Sciences and Medicine, King’s College London School of Medical Education, London, UK, david.b.hammond@kcl.ac.uk

## Main article

We were interested to read the letter of our colleagues regarding the impact Covid-19 has had on medical education [1]. That engagement levels with the online content have been high certainly seems to be the case, with virtual attendances closer to capacity than face-to-face teaching, despite some students currently being in other time-zones. The move to open-book examinations may yet be a practice that lives on, especially as one of the biggest concerns of students was finding a quiet place to sit the exam [2] – yet in a post-lockdown world, quiet – at home, or in a library, will be easier to obtain.

However, we feel it is important not to get carried away with the apparent success of online medical education. Whilst the thoroughness of online teaching may surpass other mediums, being a good doctor requires patient contact and time on the wards. Nothing else can adequately prepare someone for the realities of working life. Focusing on Paediatrics as an example, whilst a webinar can tell you how to perform a paediatric examination, only experiencing it can teach you to, sometimes, just sit on the floor and play with a child, improvising the examination as opportunities arise. Additionally, nothing can truly replace the pain and anger of parents concerned for their child – only by actually speaking to them can communication skills be honed for the situation. As Noble argued, ‘although knowledge is important, it is not what makes the difference between a good doctor and a less good doctor [3].’

Whilst the pandemic was rife and hospitals were quickly reconfigured to cope with the surge in Covid-19 patients [4], medical students on the wards may have been left unsupervised, or else diverted the attention of doctors away from patients – so education, correctly, had to temporarily take a back seat. As things begin to return to normal, however, it is vital that medical schools around the country do not think that online teaching is an acceptable substitute for clinical placements. Perhaps in the early years of medical school, mixing online lectures and webinars with face-to-face teaching may be worthwhile, in order to maintain the benefits accrued from increased real time engagement. When it comes to clinical time, however, no amount of exposure can be too great for a student and this must be protected at all costs, in order to ensure that future graduates are both well prepared and safe doctors.
